# Effect of the Combination of Levofloxacin with Cationic Carbosilane Dendron and Peptide in the Prevention and Treatment of *Staphylococcus aureus* Biofilms

**DOI:** 10.3390/polym13132127

**Published:** 2021-06-29

**Authors:** Jael Fernandez, Ángela Martin-Serrano, Natalia Gómez-Casanova, Annarita Falanga, Stefania Galdiero, Francisco Javier de la Mata, Irene Heredero-Bermejo, Paula Ortega

**Affiliations:** 1 Department of Organic and Inorganic Chemistry, and Research Institute in Chemistry “Andrés M. del Río” (IQAR), Universidad de Alcala, 28805 Madrid, Spain; yaelfc@hotmail.com (J.F.); angela.martins@uah.es (Á.M.-S.); 2 Biomaterials and Nanomedicine (CIBER-BBN), Spain and Institute “Ramón y Cajal” for Health Research (IRYCIS) Colmenar Viejo Road, Km 9, 100, 28034 Madrid, Spain; 3 Department of Biomedicine and Biotechnology, University of Alcalá, 28805 Madrid, Spain; natalia.gomezc@uah.es; 4 Department of Agricultural Science, University of Naples Federico II, Via Università 100, 80055 Portici, Italy; annarita.falanga@unina.it; 5 Department of Pharmacy, University of Naples “Federico II”, Via Domenico Montesano 49, 80134 Naples, Italy

**Keywords:** *Staphylococcus aureus*, dendron, biofilm, nanoconjugate, peptide

## Abstract

Antibiotic resistance and biofilm-related infections, persistent in conventional antimicrobial treatment, are continuously increasing and represent a major health problem worldwide. Therefore, the development of new effective treatments to prevent and treat biofilm-related infections represents a crucial challenge. Unfortunately, the extensive use of antibiotics has led to an increase of resistant bacteria with the subsequent loss of effectivity of commercial antibiotics, mainly due to antibiotic resistance and the ability of some bacteria to form microbial communities in biotic or abiotic surfaces (biofilms). In some cases, these biofilms are resistant to high concentrations of antibiotics that lead to treatment failure and recurrence of the associated infections. In the fight against microbial resistance, the combination of traditional antibiotics with new compounds (combination therapy) is an alternative that is becoming more extensive in the medical field. In this work, we studied the cooperative effects between levofloxacin, an approved antibiotic, and peptides or cationic dendritic molecules, compounds that are emerging as a feasible solution to overcome the problem of microbial resistance caused by pathogenic biofilms. We studied a new therapeutic approach that involves the use of levofloxacin in combination with a cationic carbosilane dendron, called MalG_2_(SNHMe_2_Cl)_4_, or a synthetic cell-penetrating peptide, called gH625, conjugated to the aforementioned dendron. To carry out the study, we used two combinations (1) levofloxacin/dendron and (2) levofloxacin/dendron-peptide nanoconjugate. The results showed the synergistic effect of the combination therapy to treat *Staphylococcus aureus* biofilms. In addition, we generated a fluorescein labeled peptide that allowed us to observe the conjugate (dendron-peptide) localization throughout the bacterial biofilm by confocal laser scanning microscopy.

## 1. Introduction

In the last century, infectious diseases have been caused by bacteria with specific pathogenic mechanisms and antibiotic and vaccine developments against pathogenic microorganisms have achieved a remarkable efficiency in their control. However, nowadays, the remarkable adaptation mechanisms of pathogenic bacteria have generated diverse defense mechanisms against antibiotics, which cause a severe global threat to public health [1,2,3]. Therefore, there is an urgent need to search for new antibacterial drugs.

Among the bacterial mechanisms of resistance, biofilm formation represents a remarkable implication [4]. These cell structures allow microorganisms to create a multilayer community that makes it easier for them to survive in unfavorable environments [5]. In addition, the formation of these structures is a self-defense mechanism that protects them from the innate and adaptive host immune responses and from antimicrobial treatments. In these conditions, biofilm constituting bacteria show an antibiotic resistance that is up to 1000 times greater than the planktonic cells of the same strain.

Different strategies are trying to overcome the antibiotic resistance. In this sense, new therapies are currently being searched, either with antibiotics that have high rates of susceptibility, such as levofloxacin (LEV), an agent with clinical value and a high anti-staphylococcal activity [6], or using newly synthesized molecules, such as antimicrobial peptides, which have been recently considered one of the most relevant therapeutic approaches in the treatment of biofilms [7,8,9,10]. Among these new therapeutic strategies, dendritic systems of carbosilane nature have shown to be a promising solution. Nevertheless, none of them are exempt/excluded from the disadvantages generated in consequence of their extensive use. 

However, using these systems in combination therapy may solve the disadvantages of their individual use, improving their activity because combinations may exhibit additivity or synergy, that allow a reduction in effective doses of the compounds. Only a few published works studied the combination of dendritic systems with LEV [11] or with antimicrobial peptides to date [12]. Both studies showed a synergistic effect of their antibacterial activities when using the combination against *Escherichia coli* (Gram-negative) and *Staphylococcus aureus* (Gram-positive) strains. Furthermore, we consider it of special interest to probe the consequent enhancement of the antimicrobial activity of LEV when used in combination with a cell-penetrating peptide (CPP). In this study, we used gH625, a molecule that can interact with membrane components, and transiently or locally disrupt membrane bilayers to penetrate this structure [13]. gH625 contains a high percentage of alanine, glycine and leucine residues, which are likely responsible for its conformational flexibility and ability to adopt diverse secondary structures in different environments. Moreover, these aromatic residues are involved in its preferential localization at the membrane interface [13,14,15], which may endow the molecule with the function of being an effective drug delivery carrier [16,17] without any toxic effect on mammalian cells [18,19].

Recently, a gH625 analogue with a sequence of lysine residues at its C-terminus has shown low activity against planktonic cells, while impairing the formation of polymicrobial biofilms of clinical isolates of *Candida tropicalis*/*Serratia marcescens* and *Candida tropicalis*/*S. aureus*. Likely, this analogue may influence the biofilm architecture, interfering with cell adhesion and polymeric matrix [20]. Previously, we have also probed the effect of the synergistic use of gH625 with common antibiotic molecules and found that gH625 was very effective in eradicating persistent derived biofilms alone and combined with conventional antifungals, mainly strengthening the antibiofilm activity [21].

In the present study, we focused on treating *S. aureus* biofilms that are often resistant to both antimicrobial treatments and host defense mechanisms. In consequence, this pathogen is the leading cause of numerous infections associated with medical devices that are implanted each year, causing high morbidity and mortality rates [22,23,24]. Considering the ability of cationic carbosilane dendritic systems to inhibit the formation *of S. aureus* biofilms [25], this work aims to analyze the efficiency of the combination therapy of a cationic carbosilane dendron with LEV or a CPP peptide in the prevention and eradication of *S. aureus* biofilms.

Herein, we present the anti-biofilm capacity of a cationic carbosilane dendron with a maleimide group at the focal point, their activity either in combination with LEV or the nanoconjugate formed by the carbosilane dendron and a cell-penetrating peptide, called dendron-gH625 peptide nanoconjugate (DPC). Additionally, the biofilm architecture and the viability of the cells were evaluated by confocal microscopy and scanning electron microscopy.

## 2. Materials and Methods

### 2.1. Synthesis of AcgH625C and Fluorescein-gH625C

Fmoc-protected amino acid derivatives and coupling reagents were purchased from Iris Biotech GmbH (Marktredwitz, Germany). Other chemicals (carbodiimide (DIC); [bis(dimethylamino)methylidene]({3H-[1–3]triazolo[4,5-b]pyridin-3-yl}oxidanium (HATU); Diisopropylethylamine (DIPEA); Dimethylformamide (DMF), Trifluoroacetic acid (TFA); 1,2-ethanedithiol (EDT)) were purchased from Sigma-Aldrich and Del Chimica (Milano, Italy).

The AcgH625C peptide (Ac- HGLASTLTRWAHYNALIRAFC-NH_2_) was synthesized on a Rink amide resin, p-methylbenzhydrylamine (MBHA, Iris Biotech GmbH) using the standard solid-phase Fmoc method as reported in our work [21]. Briefly, the removal of the Fmoc protecting group on the resin and amino acids was performed with a basic solution (30% *v*/*v* piperidine in DMF) for 10 min and the first coupling was carried out in the presence of 4 eq Fmoc-protected amino acid, 4 eq DIC and 4 eq oxymapure while the second coupling was carried out in presence of 4 eq amino acid, 4 eq HATU and 8 eq DIPEA. The crude peptide was divided into 2 parts. One part was acetylated using acetic anhydride solution and another part was labeled with fluorescein, the reaction was carried out overnight in the presence of HATU and DIPEA.

The obtained compounds were Acetylated-gH625-Cys-CONH_2_ (AcgH625C) and Fluorescein-gH625-Cys-CONH_2_ (F-gH625C). The crude peptides were cleaved from the resin with an acid solution (TFA/thioanisole/anisole/water/EDT 82.5/5/5/5/2.5 % vol) and precipitated in ice-cold diethyl ether, purified in a Phenomenex Jupiter 4 µm Proteo 90 Å 250 × 21.20 mm column with a linear gradient of solvent B (0.1% TFA in acetonitrile) in solvent A (0.1% TFA in water) from 20 to 80% in 20 min with UV detection at 210 nm. The peptide identities were confirmed using LTQ-XL Thermo Scientific linear ion trap mass spectrometry.

### 2.2. Carbosilane Cationic Dendron

The dendron selected to carry out this work presents a maleimide group at the focal point and positive charges on the surface. It was synthesized according to the systematic protocol described previously in our research group [12].

### 2.3. Synthesis of Carbosilane Dendron–Peptide Nanoconjugate (DPC)

**Scheme 1:**
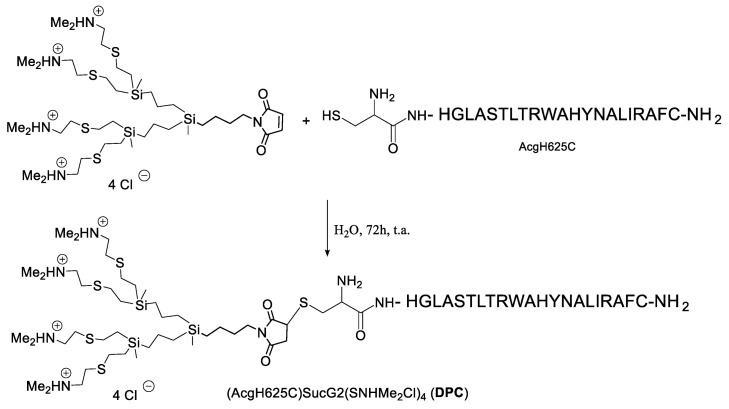
Synthesis of carbosilane dendron–peptide nanoconjugate.

A solution in distilled water of AcgH625C peptide (0.0056 g, 0.0023 mmol), previously deoxygenated with argon, was added drop by drop over the dendritic wedge MalG_2_(SNHMe_2_Cl)_2_ (0.0025 g, 0.0024 mmol) dissolved in 1 mL of water. The reaction was maintained in agitation, at room temperature and under an inert atmosphere for 24 h. Once the conjugation was completed, the solvent was evaporated under vacuum and the succinimide thioether peptide nanoconjugate (AcgH625C)SucG_2_(SNHMe_2_Cl)_4_ (**DPC**) was obtained as a brown oil (0.0077 g, 96%) (Scheme 1).

HPLC-MS analyses were performed on a Waters Alliance 2795 with an automated injector and a photodiode array detector Waters 2996 coupled to an electrospray ion source (ESI-MS) Micromass ZQ mass detector, using either a XSelectTM C18 reversed-phase analytical column (4.6 × 50 mm, 3.5 μm), or a Symmetry 300 C4 reversed-phase analytical column (4.6 × 50 mm, 3.5 μm) and the MassLynx 4.1 software. The instrument was operated in the positive ESI (+) ion mode. HPLC-MS analyses were carried out with several elution systems. System A (C18 column): a linear gradient 5–100% CH_3_CN (0.07% HCOOH) in H_2_O (0.1% HCOOH) over 4.5 min at a flow rate of 2 mL/min; System B (C4 column): a linear gradient 5–100% CH_3_CN (0.07% HCOOH) in H_2_O (0.1% HCOOH) over 30 min at a flow rate of 1 mL/min.

ESI-MS: [M + 3H]^3+^ = 1113.4 uma (Calc. 1113.2 uma), [M + 4H]^4+^ = 835.4 uma (Calc. 835.2 uma), [M + H]^5+^ = 668.6 uma (Calc. 668.4 uma), [M + 6H]^6+^ = 557.4 uma (Calc. 557.1 uma), [M + 7H]^7+^ = 478.0 uma (Calc. 477.7 uma), [M + 8H]^8+^ = 418.5 uma (Calc. 418.1 uma).

The same protocol was used to obtain the conjugate (F-AcgH625C)SucG_2_(SNHMe_2_Cl)_4_ (**F-DPC**).

### 2.4. Staphylococcus aureus: Growth Conditions and Stimulation for Biofilm Formation

*Staphylococcus aureus* strain of Colección Española de Cultivos Tipo (CECT240) was used in this study. *S. aureus* isolates were stored at −20 °C with 20% glycerol (Sigma-Aldrich, Saint Louis, MO, USA) until use. The strain was grown on Plate Counting Agar (PCA) (Scharlab, Barcelona, Spain) overnight. Then, bacteria were subcultured in Mueller Hinton (MH) (Sigma-Aldrich, Saint Louis, MO, USA) incubated with slight agitation (150 rpm) at 37 °C for 20 h to stimulate these cells to form biofilms. Protocol to form biofilms was followed as previously described [26] Bacteria were cultured in PCA petri dish at 37 °C for 24 h and then inoculated into growth medium until 0.5 McFarland units were obtained. Tubes were incubated at 37 °C for 20 h and, then, a 1:100 dilution was made with the same medium (inoculum solution). A total of 200 µL of this suspension was inoculated into a 96-well plate and incubated for 18 h at 37 °C. The ability of molecules to prevent biofilm formation (pre-treatment) and eliminate (post-treatment) *S. aureus* biofilm was studied. Antimicrobial activity assays were performed using the ISO 20776–1:2006 protocol broth microdilution reference method with reading of endpoints at 24 h.

Biofilm biomass was confirmed by staining with 1% violet crystal for 15 min. Then, the dye was removed and washed three times with PBS. The plate was dried and 200 µL of acetic acid 33% were added to remove the dye inside the cells. Then, 150 µL of the acetic acid solution were transferred into another 96-well plate to measure the absorbance of each well (640 nm).

### 2.5. Pre-Biofilm Treatment Assay

To determine the capacity to inhibit the *S. aureus* growth and prevent the development of a viable biofilm (pre-treatment), *S. aureus* was adjusted to 0.5 McFarland standard and diluted to 1:100 into Bacto Tryptic Soy Broth (TSB) supplemented with 2% glucose (Scharlab, Barcelona, Spain). To run the experiments, 96-well microtiter plates (NUNC^TM^) containing the different compounds to test (Dendron (2–128 mg/L), DCP (2–64 mg/L) or LEV (0.125–1 mg/L)) in a two-fold dilution series prepared in sterile water were inoculated with *S. aureus* cell suspension. The activity of the tested compounds against *S. aureus* cells was examined by comparison with drug-free wells (positive control). *S. aureus*-free wells containing growth medium were included (negative control). Plates were sealed with Parafilm (Bemis, Neenah, WI, USA) and incubated for 24 h at 37 °C. Assays were run in technical triplicate and repeated at least twice.

Resazurin colorimetric assay and drop plate method were performed in all experiments (shown in Section 2.8).

### 2.6. Established Biofilm Treatment Assay

The capacity of the compounds to damage and/or eliminate bacteria growth in established *S. aureus* biofilm was evaluated (post-treatment). *S. aureus* was adjusted to 0.5 McFarland standard and diluted to 1:100 into TSB supplemented with 2% glucose. Then, 100 µL of the suspension were inoculated into 96-well microtiter plates and incubated at 37 °C for 24 h. After incubation, the medium was carefully discarded, and biofilms were washed twice adding sterile PBS. Finally, serial concentrations of compounds prepared in TSB and 2% glucose were added to a final volume of 100 μL (Dendron 16–512 mg/L, DPC 16–512 mg/L or LEV 1–512 mg/L). A drug-free control (positive control) and un-inoculated control containing growth medium (negative control) were included in all experiments. Plates were sealed with Parafilm^®^ and incubated for 24 h at 37 °C. Assays were run in technical triplicate and repeated at least twice.

Resazurin colorimetric assay and drop plate method were performed in all experiments (shown in Section 2.8).

### 2.7. Combined Activity Assay

The combined activity was studied against *S. aureus* cells using the microdilution checkerboard method [27]. The inhibition of biofilm formation and the efficacy to eliminate viable cells in established biofilms were tested. The antibiotic LEV was used in combination with dendron or DPC (dendron (MalG_2_(SNHMe_2_Cl)_2_) + peptide (AcgH625C)).

For pre-biofilm treatments, LEV concentrations ranging from 0.125 to 0.25 mg/L were used. On the other hand, concentrations of 8, 16 and 32 mg/L of dendron alone and 4, 8, 16, 32 and 64 mg/L of DPC were used in this study in the presence of LEV. However, to study the ability to eliminate the established biofilms (post-treatment) 2, 4 and 8 mg/L of LEV were used, while concentrations of 64, 128 and 256 mg/L of dendron and DPC were used. Plates were sealed with Parafilm^®^ and incubated for 24 h at 37 °C. Assays were run in technical triplicate and repeated at least twice.

Resazurin colorimetric assay and drop plate method were performed in all experiments (shown in Section 2.8).

### 2.8. Resazurin Colotimetric Assay

Resazurin colorimetric assays were used in all the experiments to assess *S. aureus* viability after individual and combined treatments. Metabolically active bacteria reduce the resazurin dye. After treatment and incubation, each well was washed twice with PBS and wells were filled with 100 μL of PBS. Then, 20 μL of resazurin solution (Sigma-Aldrich), prepared at 0.01% (*w*/*v*) in sterile distilled water and filtered through a 0.22 μm pore-size filter [28], were added to each well. An un-inoculated control containing PBS was included (blank). Plates were incubated in the dark for 24 h at 37 °C. Absorbance was measured in a microplate reader (EpochTM, BioTek) at 570 and 600 nm.

This assay determined the lowest concentration that completely inhibited the *S. aureus* growth and, in consequence, the biofilm formation (not metabolic activity observed/not dye reduction observed). It was denominated as the minimum biofilm inhibitory concentration (MBIC) in pre-biofilm treatment assay [28]. Using this method, we also determined the minimum biofilm damaging concentration (MBDC) in post-biofilm treatment assay, defined as the lowest concentration that caused cell damage by inhibiting the established biofilm of *S. aureus*. At this concentration, there was no observed absorbance signal with the colorimetric assay.

Viability percentages were calculated comparing values to the non-treated controls included in all experiments.

### 2.9. Drop Plate Method

The minimum bacterial concentration (MBC) value for the pre-treatment assay, the lowest concentration that eliminated 100% of the cells stimulated to generate a biofilm, and the minimum biofilm eliminating concentration (MBEC) value for the post-biofilm treatment assay, the lowest concentration capable of completely eradicating bacteria from the previously established biofilm, were obtained by the drop plate method observing no growth on agar plates [28,29,30,31]. To perform these experiments, wells were scraped with the pipette tips to detach the biofilm cells and 5 μL of each well suspension were transferred to PCA agar plates and incubated overnight at 37 °C.

### 2.10. Confocal Laser Scanner Microscope

To observe the effect of the studied compound, confocal laser scanning microscopy (CLSM) was used. CLSM assays were performed to evaluate the viability of *S. aureus* biofilms grown on glass coverslips. *S. aureus* biofilms were washed with PBS and stained using 1% propidium iodide (PI) (Merck KGaA, Germany). Plates were incubated in the dark for 15 min. Dead cells or cells with membrane damage were stained in red. The fluorescein-gH625C peptide was covalent conjugated as previously described above (Section 2.3). This fluorescein group allowed the peptide to be observed in the green channel. Stained biofilms were observed with a LEICA TCS-SL or SP5 Confocal Laser Scanning Microscope, using argon and helium/neon ion lasers. The excitation/emission range for PI is 490/635 nm. Non-treated controls were included in all experiments.

### 2.11. Scanning Electron Microscopy

Scanning electron microscopy (SEM) was performed to evaluate biofilm alterations after treatments. *S. aureus* was grown on a glass coverslip, as indicated in Section 2.6 to form biofilm structure, and then fixed in Milloning’s solution containing 2% glutaraldehyde for 24 h. After incubation time, cells were washed in Milloning’s solution with 0.5% glucose and dehydrated first through an ethanol series and finally with anhydrous acetone. Samples were critical-point dried using a Polaron CPD7501 critical-point drying system, and sputter-coated with 200 ’Å gold-palladium using a Polaron E5400. Scanning electron microscopy was performed at 5–15 kV in a Zeiss DSM 950 SEM

### 2.12. Statistical Analysis

GraphPad Prism 8.4 program for Windows (GraphPad Software, 2020, San Diego, CA, USA) was used to analyze all data. ANOVA and Tukey’s test were performed, considering *p* < 0.05 values as statistically significant.

## 3. Results and Discussion

One of the objectives of the combination therapy is to administer pharmacological agents that have a different mode of action and target different cell structures to increase their effectiveness and eliminate the generation of resistances. For this reason, systems with different modes of action were selected to perform this study.

The antibacterial activity mode of cationic carbosilane dendritic systems can be ascribed to the interaction of their positive charges with the negatively charged bacterial membrane. This interaction results in the displacement of divalent ions allocated on the surface, such as calcium and magnesium, leading to membrane disruption and cell death [25]. On the other hand, LEV belongs to the fluoroquinolone drug class and exerts its antimicrobial activity via the inhibition of two critical bacterial enzymes: DNA gyrase and topoisomerase IV [32]. In the case of the selected gH625 peptide, a CPP [20], its mode of action has not been entirely reached yet. However, it has been described that it does not form pores in the bacterial membrane, but it is likely to act on the structure of the biofilm. For this reason, it may have a low activity against planktonic cells and a moderate activity in the treatment of biofilms [21].

### 3.1. Formation of Nanoconjugate: Dendron-gH625 Peptide (DPC)

To carry out this work, we selected a second-generation carbosilane dendron, MalG_2_(SNHMe_2_Cl)_4_, with a maleimide group at the focal point and four positive charges, due to the presence of four ammonium units on its surface. This selection was made considering its demonstrated ability to bind peptides through thioether bonds and its antibacterial activity in *S. aureus* planktonic cells [12]. On the other side, to conjugate the peptide in a covalent manner to the dendritic skeleton, it was necessary to modify the gH625 peptide sequence by adding an extra C-terminal cysteine residue that provides the thiol group needed for conjugation to the maleimide focal point of previously synthesized dendrons (Scheme 1). The nanoconjugate dendron-peptide AcgH625CSucG_2_(SNHMe_2_Cl)_4_ (DPC) was formed in water as a solvent and its formation was corroborated by HPLC-MS.

### 3.2. Individual Activity against S. aureus Biofilm Formation and Established Biofilms

#### 3.2.1. Efficacy of New Dendritic Molecules Inhibiting Biofilm Formation of *S. aureus* (Pre-Treatment)

Firstly, it was necessary to establish the active dose of individual compounds to inhibit *S. aureus* growth (MBIC) and prevent biofilm formation (MBC) (pre-treatment). In addition, establishing the active dose to damage (MBDC) and eradicate (MBEC) bacteria from established *S. aureus* biofilms was also necessary (post-treatment). These data were required to determine the existence of a cooperative effect between different drugs. To perform these experiments, we studied the activity of the dendron and the DPC. In the pre-treatment, we determined a MBIC of 64 mg/L for the dendron (MBC of 128 mg/L), while for the DPC we only obtained a 25% reduction in biofilm cell viability of *S. aureus* at the same concentration (*p* < 0.05) (Table 1). However, 64 mg/L of the DPC (corresponding to 19.12 mg/L of the dendron within the nanoconjugate) reached the same reduction in viability as 16 mg/L of the dendron alone. Therefore, our data indicated that the anchoring of the peptide to the dendron by tiol-en coupling reaction does not reduce the dendron’s antibacterial activity.

#### 3.2.2. Efficacy of New Dendritic Molecules Removing or Damaging Established Biofilms of *S. aureus* (Post-Treatment)

Biofilm formation has tremendous clinical implications because these microbial communities act as a reservoir of pathogenic cells. These biofilms also provide antimicrobial resistance to high concentrations of antibiotics due to the low metabolic rate of the cells that form it, and their ability to act as a barrier, making it difficult for antimicrobial agent to penetrate inside of them. Our data showed that at 16 mg/L of the dendron, the viability of *S. aureus* was reduced by 25% in the pre-treatment. However, the viability was only reduced by 10% using dendron alone at the same concentration against established biofilms (post-treatment) (*p* < 0.05). Even using the DPC at 32 mg/L, the same activity against biofilm cells was achieved using a lower amount of dendron (9.56 mg/L of dendron in 32 mg/L DPC). On the other hand, it is important to remark that the gH625 peptide did not have any activity by itself. Therefore, our data indicated that the presence of gH625 peptide would facilitate the dendron entry into the biofilm and improve its activity against biofilm cells. It is probably due to its ability to interact with phospholipid groups of the bacterial membrane as a consequence of the aromatic residues present in peptide structure and its conformational structural plasticity [20]. However, biofilm eradication was not achieved at any of the concentrations tested in these post-treatment assays (Table 1).

### 3.3. Combined Therapy Assays

The efficacy of the combined treatments, dendron-LEV and DPC-LEV, was evaluated in the inhibition of *S. aureus* growth and prevention of biofilm formation (pre-biofilm treatment), as well as against the eradication of bacteria from a previously established biofilm (post-biofilm treatment), that was determined as the ability to produce death of the bacteria embedded in the biofilm once it is established. Results are described below. A colorimetric assay, resazurin, was used to assess cell viability of the bacteria embedded in these structures after treatments.

#### 3.3.1. Pre-Treatment: Dendron-LEV vs. DPC-LEV Combination

Our data showed significant differences in biofilm pre-treatment when we studied the effect of combination dendron-LEV or DPC-LEV. In the first combination, Dendron-LEV, the viability was reduced by 54% using 16 mg/L of the dendron (MBIC = 64 mg/L) and 0.25 mg/L of LEV (MBIC = 0.5 mg/L), and using the same concentration of LEV in combination with 32 mg/L of dendron the reduction reached increase by 65% at (*p* < 0.05). Reducing the amount of LEV present in the combination to 0.12 mg/L, a slight decrease of the viability with the dendron was only noted at 32 mg/L.

However, in the case of combination DPC-LEV it was possible to observe a better effect. When the LEV concentration used was 0.25 mg/L the viability was reduced by 50% and 65% in the presence of 16 mg/L of the DPC (4.78 mg/L of dendron present in the conjugate DCP) or 32 m/L of the DPC (9.56 mg/L of dendron present in the conjugate DCP), respectively (Table 2). Using a lower amount of LEV (0.12 mg/L), the viability was reduced by 57% in the presence of 32 mg/L of DPC (9.56 mg/L of dendron).

These results indicated a cooperative effect between the dendron or the DPC in combination with LEV. Therefore, the combined therapy reduced the amount of dendron or DPC needed to inhibit biofilm formation on their own to at least one third. In addition, it is important to point out that the combination DPC-LEV was more effective than the combination Dendron-LEV. Comparing the viability obtained by combining 0.25 mg/L of LEV and 8 mg/L of dendron (viability 87%) to 0.25 mg/L of LEV and 16 mg/L of DPC (viability 53%, 9.56 mg/L of dendron present in the conjugate DCP, the same concentration of dendron), the results indicated that the peptide presence makes the nanoconjugate more active. Additionally, in combination with antimicrobials, as in the case of LEV (DPC-LEV), its efficacy increases significantly, and it is twice as effective at inhibiting the formation of biofilms than DPC without LEV combination.

#### 3.3.2. Post-Treatment of Biofilm: Dendron-LEV vs. DPC--/LEV Combination

Our data showed significant differences in the biofilm post-treatment when combining the dendron with LEV. In general, and looking at the data obtained in Table 3, it could be observed that at the low concentrations of DPC used in this study there was a small inhibition of cell viability in combination with 2 mg/L of LEV, unlike using dendron alone. The behavior of the dendron and the DPC was similar in the presence of 4 mg/L of LEV. The best combination found was 8 mg/L of LEV with different concentrations of DPC. This combination was more effective than the combinations that used other concentrations of LEV. However, the activity of the dendron in the presence of 8 mg/L LEV was not only worse than the DPC activity, but also it was worse than the activity reached with the 4 mg/L LEV combination. In the dendron-LEV combination, LEV may be responsible for removing part of the biofilm, since at individual experiments we observed that the dendron was unable to eradicate the biofilm bacteria completely (only 10% non-viable cells observed) and LEV did not show more activity by itself. It could also be observed that the treatment showed even worse activity at higher concentrations (Table 3). This fact may be probably due to the faster and deeper internalization of the dendron inside the phospholipid bilayer present in the bacterial membrane, which, consequently, impedes the entry of LEV into the biofilm [12].

However, it could be seen that the effectiveness of DCP is more remarkable, reducing the viability of an established biofilm by 40% using 256 mg/L of DCP (76.48 mg/L of dendron present in the conjugate DCP) and 8 mg/L of LEV (*p* < 0.05). These findings may indicate that the presence of the gH625 peptide facilitates the entry of the dendron into the biofilm. The DPC and LEV showed a lower activity individually than at the concentrations mentioned for the combination (10% and 20% non-viable cells, respectively). Additionally, when DCP and LEV were administered in combination, it was possible to observe that both compounds were able to increase their activity, which shows a combined effect between them due to gH625 peptide presence (Table 3). In addition, the combinatory therapy of dendron and LEV did not increase the activity. This fact reassured that peptide presence is responsible for activity improvement. Nonetheless, the complete biofilm bacteria eradication was not achieved at any concentration. These results were determined because bacteria growth was observed on agar after plating well suspensions.

### 3.4. Confocal Microscopy

**Figure 1:**
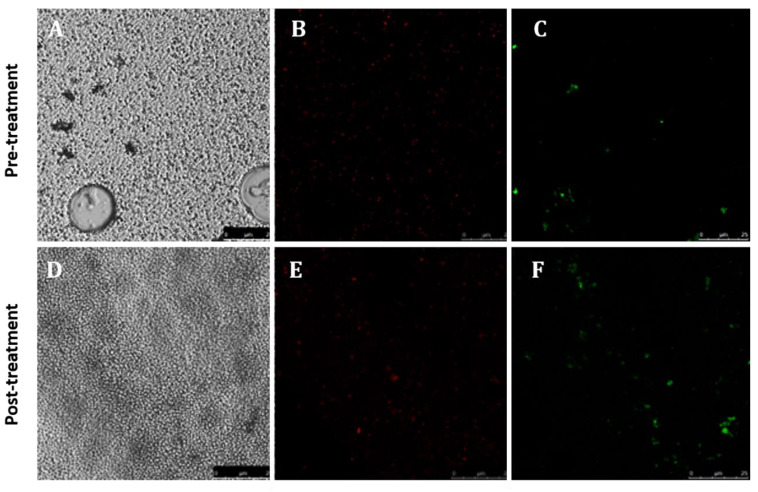
Confocal electron microscopy images of pre- and post-treatment using 32 mg/L of F-DPC ((F-gH625C)SucG_2_(SNHMe_2_Cl)_4_) (**2**). (**A**) Treated biofilm structure (phase), (**B**) PI signal: dead cells (red channel), (**C**) F-gH625C signal (green channel), (**D**) treated biofilm structure (phase), (**E**) PI signal: dead cells (red channel), (**F**) F-gH625C signal (green channel).

These experiments aimed to determine the activity of the nanoconjugate (DPC). The concentration selected for these experiments was 32 mg/L of nanoconjugate (F-DPC). In these treatment conditions low numbers of non-viable cells would be observed, and the biofilm structure would remain unaltered in the pre- and post-treatment experiments. Firstly, it is important to point out that the treatments with the F-gH625C peptide alone showed the absence of red fluorescence of the bacteria by PI in the images obtained for pre- and post-treatment, which further supports that the peptide does not cause damage over planktonic, or bacteria embedded in the biofilm. This result confirmed the peptide ability to penetrate into the biofilm without causing alterations on it. When the treatment was carried out with F-DCP, the confocal images showed that after the treatment some bacteria appear stained with red dye (Figure 1B,E pre- and post-treatment, respectively). These observations indicated that compounds altered the cell wall and plasma membrane, that resulted in the disruption of the permeability barrier of microbial membrane structures and allowed the entry of PI dye (cell death). Additionally, the accumulation of fluorescence (green channel) in some areas of the biofilms suggested the formation of aggregates [33] (Figure 1C,F pre- and post-treatment, respectively), which may be due to the ability of the peptide to form peptide oligomer “bridges” between neighboring bacteria acting as a kind of “glue” between bacteria [34,35]. On the other hand, the treatment of established biofilms with the F-DPC reveals its biocidal action. The activity of these types of carbosilane cationic compounds has been associated with the damage of the cytoplasmic membrane of bacteria [25]. The membrane is an indispensable organelle for bacteria survival, as it contains proteins and holds many vital processes [36]. Therefore, these compounds may damage the cell membrane and may interfere with essential cellular functions.

### 3.5. Scanning Electron Microscopy (SEM)

**Figure 2:**
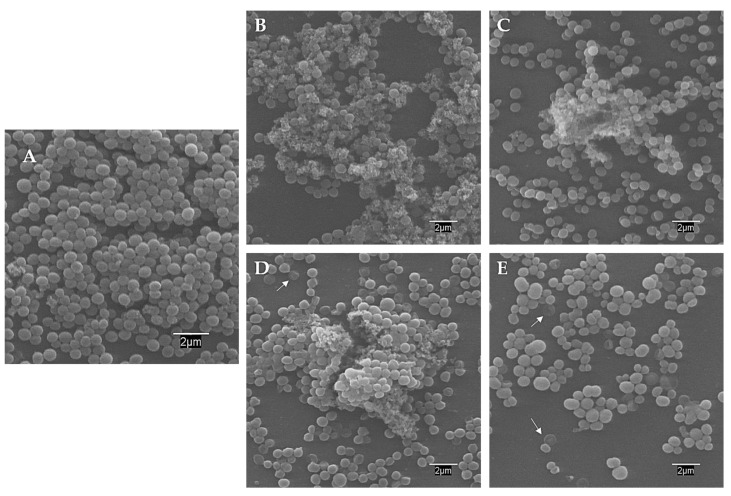
SEM images of post-treatment alteration produced on *S. aureus* biofilms. (**A**) Untreated control; (**B**) 64 mg/L dendron; (**C**) 64 mg/L DPC; (**D**) 64 mg/L dendron- 8 mg/L LEV (Dendron-LEV); (**E**) 64 mg/L DPC- 8 mg/L LEV (DPC-LEV). White arrows: collapsed cells.

To further study the effects of treatments on the biofilms, the morphology of untreated and treated *S. aureus* biofilms was visualized using SEM. These observations revealed a typical homogeneous and dense biofilm in the control samples, with different cell layers of rounded-shape cells with normal smooth surface that formed the biofilm structure (Figure 2A). However, biofilm density was reduced after treatments and dispersion between biofilm cells was observed (Figure 2B–E). We observed more individual cells. Additionally, treated biofilms showed alterations to *S. aureus* cell morphology: collapsed cells (Figure 2D,E. Arrows). These observations validated the results obtained from resazurin assays and showed the inhibitory effect and biofilm eradication activity of the compounds studied against *S. aureus* biofilm.

## 4. Conclusions

Combination therapy is considered one of the main strategies to address the problem of antimicrobial resistance to conventional antibiotics. In this contribution, two different approaches were proposed for the inhibition and treatment of *S. aureus* biofilms. The results showed that the combinations of levofloxacin with a cationic carbosilane dendron MalG_2_(SNHMe_2_Cl)_4_ or the conjugate AcgH625CSucG_2_(SNHMe_2_Cl)_4_ (DPC) are effective for preventing biofilm formation and treating established biofilms at concentrations that when tested individually they achieved low efficiencies. Therefore, they showed antibiofilm damaging and antibiofilm inhibitory activities. Furthermore, the gH625 peptide included in the conjugate (DPC) increases the dendron’s efficiency by reducing the amount of dendron needed to inhibit biofilm viability by at least one third in biofilm formation treatments or established biofilm treatments. In consequence, the highest percentages of inhibition were obtained with the DPC-levofloxacine combination. In conclusion, these preliminary in vitro studies results may suggest that the DPC combination with levofloxacin would be an interesting alternative to treat and control biofilm-associated infections, particularly those with reduced susceptibility to most of the recommended antibiotics

## Figures and Tables

**Table 1:** MBIC, MBC, MBDC and MBEC values of dendron and DCP against *S. aureus* biofilm formation (pre-treatment) and established biofilms (post-treatment).

	Pre-Treatment		Post-Treatment	
	MBIC (mg/L)	MBC (mg/L)	MBDC (mg/L)	MBEC (mg/L)
Dendron	64	128	>512	NE
DPC ^1^	>64	>64	>512	NE

NE: not biofilm eradication. Growth observed on PCA. ^1^ Concentration of dendron in each DPC concentration: 64 mg/L (19.12 mg/L of dendron), 128 mg/L (38.24 mg/L of dendron), 512 mg/L (152.96 mg/L of dendron).

**Table 2:** Percentage of non-viable biofilm cells for the different combinations of Dendron-LEV vs. DPC-LEV in the pre-treatment of *S. aureus* biofilm (inhibition of biofilm formation).

**PRE-TREATMENT**		**Dendron-LEV**			
		**LEV 0.25 mg/L**	**Not viable % (mean ± SD)**	**LEV 0.12 mg/L**	**Not viable %** **(mean ± SD)**
	**Dendron** **(mg/L)**	32	65 ± 9.01%	32	30 ± 6.58%
		16	54 ± 3.79%	16	0 ± 0.00%
		8	13 ± 3.11%	8	1 ± 4.08%
		**DPC-LEV**			
		**LEV 0.25 mg/L**	**Not viable % (mean ± SD)**	**LEV 0.12 mg/L**	**Not viable %** **(mean ± SD)**
	**DPC ^1^** **(mg/L)**	32	65 ± 9.77%	32	58 ± 4.06%
		16	47 ± 9.28%	16	38 ± 4.72%
		8	27 ± 1.99%	8	22 ± 7.79%
		4	11 ± 4.51%	4	9 ± 9.26%

SD: standard deviation values. ^1^ Concentration of dendron in each DPC concentration: 32 mg/L (9.56 mg/L of dendron), 16 mg/L (4.78 mg/L of dendron), 8 mg/L (2.39 mg/L of dendron), 4 mg/L (1.19 mg/L of dendron).

**Table 3:** Percentage of non-viable biofilm cells for the different combinations of Dendron-LEV vs. DPC-LEV in the post-treatment of *S. aureus* biofilm (inhibition of established biofilm).

**POST- TREATMENT**		**Dendron-LEV**					
		**LEV** **8 mg/L**	**Not viable % (mean ± SD)**	**LEV** **4 mg/L**	**Not viable % (mean ± SD)**	**LEV** **2 mg/L**	**Not viable % (mean ± SD)**
	**Dendron** **(mg/L)**	256	13 ±3.05%	256	26 ± 13.01%	256	12 ± 6.14%
		128	12 ± 7.40%	128	25 ± 3.39%	128	0 ± 0.00%
		64	12 ± 6.8%	64	23 ± 4.92%	64	0 ± 0.00%
		**DPC-LEV**					
		**LEV** **8 mg/L**	**Not viable % (mean ± SD)**	**LEV** **4 mg/L**	**Not viable % (mean ± SD)**	**LEV** **2 mg/L**	**Not viable % (mean ± SD)**
	**DPC ^1^** **(mg/L)**	256	41± 4.28%	256	27 ± 9.27%	256	14 ± 8.06%
		128	36 ± 4.91%	128	28 ± 10.09%	128	8 ± 5.38%
		64	36 ± 8.76%	64	34 ± 10.49%	64	3 ± 2.01%

SD: standard deviation values. ^1^ Concentration of dendron in each DPC concentration: 64 mg/L (19.12 mg/L of dendron), 128 mg/L (38.24 mg/L of dendron), 256 mg/L (76.48 mg/L of dendron).

## Data Availability

Not applicable.
